# Child maltreatment, cognitive functions and the mediating role of mental health problems among maltreated children and adolescents in Uganda

**DOI:** 10.1186/s13034-021-00373-7

**Published:** 2021-04-30

**Authors:** Herbert E. Ainamani, Godfrey Z. Rukundo, Timothy Nduhukire, Eunice Ndyareba, Tobias Hecker

**Affiliations:** 1 Department of Mental Health, Kabale University School of Medicine, Kabale, Uganda; 2 Department of Psychology, Bishop Stuart University, Mbarara, Uganda; 3 Department of Psychiatry, Mbarara University of Science and Technology, Mbarara, Uganda; 4 Department of Pediatrics, Kabale University School of Medicine, Kabale, Uganda; 5 Department of Educational Psychology, Kabale University, Kabale, Uganda; 6 Department of Psychology, Bielefeld University, Bielefeld, Germany

**Keywords:** Child maltreatment, Cognitive functions, Mental health, East Africa

## Abstract

**Background:**

Child maltreatment poses high risks to the mental health and cognitive functioning of children not only in childhood but also in later life. However, it remains unclear whether child maltreatment is directly associated with impaired cognitive functioning or whether this link is mediated by mental health problems. Our study aimed at examining this research question among children and adolescents in Uganda.

**Methods:**

A sample of 232 school-going children and adolescents with a mean age of 14.03 (*SD* = 3.25) was assessed on multiple forms of maltreatment using the Maltreatment and Abuse Chronology Exposure—Pediatric Version (pediMACE). Executive functions were assessed by the Tower of London task and working memory by the Corsi Block Tapping task, while mental health problems were assessed using the *Child PTSD Symptom Scale for PTSD and the Center for Epidemiological Studies Depression Scale* for Children (CES-DC).

**Results:**

In total, 232 (100%) of the participant reported to have experienced at least one type of maltreatment in their lifetime including emotional, physical, and sexual violence as well as neglect. We found a negative association between child maltreatment and executive functions (β = − 0.487, *p* < 0.001) and working memory (β = − 0.242, *p* = 0.001). Mental health problems did not mediate this relationship.

**Conclusions:**

Child maltreatment seems to be related to lower working memory and executive functioning of affected children and adolescents even after controlling for potential cofounders. Our study indicates that child maltreatment the affects children’s cognitive functionality beyond health and well-being.

**Supplementary Information:**

The online version contains supplementary material available at 10.1186/s13034-021-00373-7.

## Background

Child maltreatment which is defined as any act of abuse or neglect by a parent, caregiver or a community member that results in harm, potential harm, or threat of harm to a child has been remarked as one of the greatest global public health concerns [1]. Child maltreatment may include, emotional, physical, and sexual violence as well as neglect [1–3]. Estimates on the prevalence of violence against children in low- and middle-income countries (LMICs) show that a minimum of 50% of children in Asia and Africa between the ages of 2 and17 experience violence in their upbringing [4]. Various studies in Africa have shown that there is high level of child abuse in varying samples of children and adults [5]. Child maltreatment, has also been documented in East-African families, e.g., in Tanzania more than 90% of the children reported to have experienced violent discipline by parents [6] and different forms of maltreatments by teachers [7].While in Kenya, severe forms of child sexual abuse was reported [8] with key perpetrators being relatives (29%).

Similarly, studies in Uganda have reported that children and adolescents experienced violence very frequently [9–11]. For example, physical and emotional violence have been experienced by 98% of children, sexual violence by 76% and economic violence by 74% [10].

Child maltreatment does not only inflict physical pain on the affected children but also poses a major risk to cognitive impairment in both childhood and adulthood [12–14]. In support of the above findings, the toxic stress theory implicates exposure to early childhood adversity for altering the neuro-endocrinal immune system, which renders individual vulnerability to all forms of functional impairments and disease [15]. A systematic review of cognitive function after childhood trauma concluded that cognitive abnormalities may be linked to neuro-psychological and neurological impacts [16]. Studies with both animals and humans show that exposure to adverse experiences in early life affects brain regulation and endocrine responses to stress [17–19]. In fact, a number of studies on neuropsychological impairments have observed a significant disruption in prefrontal cortex that plays an important role in executive functioning following exposure to trauma and subsequent PTSD diagnosis [20–25]. Moreover, previous studies have documented how exposure to continuous stress affects hippocampal volume resulting from constant increase in glucocorticoid hormone which is released as the brain seeks to mitigate negative effects of stress. Stress in turn seems to affect the functionality of human memory and learning [18, 26–28].

Therefore, it is not surprising that a systematic review on the impact of child maltreatment on later cognitive functions reported that children with experiences of child maltreatment performed poorly on tasks of working memory, attention, episodic memory and executive functions [29]. In line with these findings, other studies mostly from high income countries showed that as a consequence of adverse childhood experiences many affected children and adolescents suffer from cognitive deficiencies [25, 30, 31]. For example, child abuse was found to be associated with delayed language development, cognitive development, and a lower intelligence quotient [12, 32]. A systematic review indicated that maltreated children performed poorly on tasks requiring executive functions, working memory and attention [33].

In addition to the high risk of cognitive impairment associated with child maltreatment, studies in high income countries have also found a strong association between maltreatment and mental health problems [34–36]. Others have repeatedly found links between maltreatment, PTSD, depression, impairments of prefrontal cortex functioning and dysregulation of HPA [37–40]. Furthermore, a negative effect of PTSD symptoms severity on cognitive functions but not trauma, has been frequently reported [24, 41, 42].

The described association between maltreatment and impaired cognitive functioning, maltreatment and mental health problems, as well as mental health problems and impaired cognitive functioning raise the question whether the association between child maltreatment and cognitive functions may be mediated by mental health problems [12, 43]. A review of maltreatment studies recommended further studies that control for the mediation effect of psychiatric co-morbidities [32]. In line with this recommendation and other previous findings, it remains unclear whether child maltreatment is directly associated with impaired cognitive functioning or whether this link is mediated by mental health problems. Moreover, most of the studies testing this link have been conducted in high income-countries. Only one study in Tanzania showed that the relation between child maltreatment and cognitive functioning was mediated by internalizing mental health problems. The other existing studies in low- and middle-income countries, in which violence against and maltreatment of children is much more common, have either examined cognitive impairment among people living with HIV [44–46], refugees [41] and the elderly [47] but not among children and adolescents with a history of maltreatment.

To close this gap, our study sought to examine the relationship between child maltreatment and cognitive functioning (working memory and executive functions) and the mediating role of mental health problems (PTSD symptom severity and depressive symptoms) among maltreated children and adolescents in Southwestern Uganda. We hypothesized that child maltreatment would be negatively correlated with (a) working memory and (b) executive functions, and (c) that these relations would be mediated by mental health problems.

## Methods

### Participants

In total, 232 children and adolescents (52% male) with a mean age of 15 years (*SD* = 2.95) participated in our study. Overall, boys were older than girls and the majority (*n* = 145, 63%) attended primary school (Table 1). In total, 101 (44%) participants were living with their mothers as primary caregivers, 40 (17%) were under the primary care of their grandparents, 21 (9%) were primarily cared for by their fathers and 21(9%) were cared for by their siblings, while 30 (13%) indicated other relatives as their main primary caregivers. Only 19 (8%) participants were cared for by another person. Overall, 103 (44%) participants reported to have lived in more than two families in their lifetime.

**Table 1:** Descriptive statistics of the main study variables and gender differences

	Male (n = 112)		Female (n = 120)		t
	M	SD	M	SD	
Age	14.74	2.95	13.38	3.35	3.29*
Education	6.76	2.28	6.02	2.54	2.34
Trauma load	4.95	1.98	5.73	2.37	− 2.70
PTSD symptoms severity	34.17	16.19	35.43	15.44	− 0.60
Depressive symptoms	24.68	10.84	27.31	9.80	− 1.94
Working memory	6.16	2.46	6.26	2.40	− 0.31
Executive functions	17.9	7.67	17.4	8.07	0.47
Maltreatment	22.35	6.41	24.47	7.55	− 2.30*
Composite score of mental health problems	− 0.17	1.87	0.16	1.66	− 1.44

*t* t-test or Welch-test statistics

*p < 0.01

### Study setting and design

This was a cross-sectional study in which 232 children and adolescents between the ages of 8 to 18 studying at two primary schools and one secondary school located in the districts of Mbarara and Rubanda in Southwestern Uganda. The three schools were chosen because they were mainly supported by nonprofit organizations and were expected to have enrolled at-risk children in terms of maltreatment experiences. On average, the enrollment per each school was at 300 (n = 900) children with a total number of 305 children recorded as the most at risk of child maltreatment. Southwestern Uganda is mainly inhabited by Bantu and Nile Hamites ethnic groups. Most residents live in rural areas, where the local economy is primarily characterized by subsistence economy with high levels of food and water insecurity [48, 49].

### Recruitment and sampling procedure

Data were collected between June 2018 and May 2019. The social workers within the schools helped to locate children and adolescents with a known history of maltreatment in their schools. Only children below the ages of 18 were recruited. We interviewed all children that were identified by the school social workers until there were no more potential participants. Two counsellors and one psychologist conducted the interviews.

The interviewers went through 1-week training in the psychological assessment and practiced the assessment in joint interviews to accomplish high inter-rater reliability. Generally, each interview took 45–60 min in a private setting within the school premises.

### Ethical considerations

Ethical clearance was obtained from the Mbarara University of Science and Technology Research Ethics Committee (MUST-REC) under approval number 07/02-18 and the Uganda National Council for Science and Technology (UNSCT) under approval number SS 4928.

Additionally, we sought permission from the school Head Teachers who introduced us to the school social workers. Before the interviews, information on the content, procedures, risks, the right to withdraw, and confidentiality were explained to the participants. Written informed consent (signature or fingerprints) of the legal guardian were obtained. In addition, children and adolescents provided their assent before participating in the study. Each family received two bars of soap as a token of appreciation for taking part in the study. Children with severe symptoms of mental health problems were referred to the nearest health facilities for specialized psychological treatment.

### Measures

All instruments were translated into Runyankole-Rukiga and back translated to English in a blind written form to ensure the original meaning was not lost. Face to face interviews included socio-demographic information, such as age, gender, educational level and having stayed in two or more homes.

### Child maltreatment

Child maltreatment and other adversities encountered at home were assessed using the Maltreatment and Abuse Chronology of Exposure—Pediatric Version (pediMACE). This tool was the child-appropriate version of the Maltreatment and Abuse Chronology of Exposure [50]. The pediMACE consists of 45 dichotomous (yes/no) questions, measuring witnessed or self-experienced forms of childhood maltreatment throughout one’s lifetime. We summed up all the questions to a total child maltreatment score (possible range: 0 to 45) that was subsequently used in the analysis.

### Exposure to traumatic events

Exposure to traumatic events was assessed using a 15-item checklist on the revised version of the Child PTSD Symptoms Scale for DSM-5—Self-Report (CPSS-VSR) [51]. With response to yes or no, children were assessed on their exposure to severe natural disasters, severe accidents, being robbed or threatened, being slapped or knifed, seeing relative being beaten or slapped and many others. The scale provides the participants with an opportunity to mention any other traumatizing event that could have been experienced. We summed up all the 15 items to come with a total score that was subsequently used in the analysis.

### Mental health problems

We used the CPSS-VSR also to assess the PTSD symptoms severity [51]. This is a 20-item scale that assesses the occurrence and frequency of PTSD symptoms in relation to the most distressing event experienced by an individual. Participants were asked to rate the frequencies of listed symptoms during the previous 2 weeks on a 5-point Likert scale from 0 (not at all/only once) to 4 (almost every week). A total sum score was calculated. A cut off score of 31 indicated a probable PTSD diagnosis [52]. This scale has good psychometric measures and showed good psychometric properties, e.g. a Cronbach alpha of 0.92 and test retest reliability of r = 0.93 [53]. In the current study the Cronbach alpha was 0.86.

Furthermore, we assessed for depressive symptoms using the Center for Epidemiological Studies Depression Scale for Children (CES-DC) [54]. This is a 20-item self-report depression inventory with total scores ranging from 0 to 60. Each response to an item is scored as: 0 = “Not at All” 1 = “A Little” 2 = “Some” 3 = “A Lot”. Items 4, 8, 12, and 16 are phrased positively, and thus were inverted prior to the calculation of the total score. Higher CES-DC scores indicate higher levels of depression. Scores above 15 have been suggested to indicate significant depressive symptoms in children and adolescents [55]. However, in this study, we used an adopted cut-off score [56]. Owing to the cultural context in East Africa, the authors set the cut-off point of probable depression at > 30 [53]. In the Rwandan study, CES-DC was validated and showed good psychometric properties: Cronbach alpha of 0.86 and test–retest reliability of r = 0.85. In the current study, the Cronbach alpha was 0.87.

### Working memory capacity

Corsi-block tapping (CBT) task was used to assess working memory. This neuropsychological test, which has been widely used as a measure of spatial memory in both clinical and experimental contexts for several decades and comes with good psychometric properties shown in validation studies [57, 58]. It has also been successfully used in studies within the Great Lakes Region in Central Africa [7, 41].The task requires participants to reproduce block-tapping sequences of increasing length in the same or in the reversed order and provides an index of working memory capacity. The Corsi apparatus consisted of nine 2.25 cm^3^ black, wooden blocks fixed to a 27.5 cm × 22.8 cm grey, wooden board. The blocks were placed as described in the original test developed by Corsi [58, 59]. Each cube was numbered on one side so that the numbers were visible to the interviewer but not to the participant. The participant was seated in front of the interviewer, who subsequently tapped the blocks starting with a sequence of three blocks. Three trials were given per block sequence of the same length. The blocks were touched with the index finger at a rate of approximately one block per second with no pauses between the individual blocks. In the first application of the test after the first half of the interview, the participant had to tap the block sequences in the same order immediately after the interviewer was finished. In the second application at the end of the interview, the participants had to tap the block sequence in reversed order. We computed a total score for both applications (same order and reversed order) by adding the number of correctly repeated sequences until the test was discontinued (i.e., the number of correct trials). The total score ranged from 0 to 21. High performance implied higher working memory capacity.

## Executive functions

The Tower of London (TOL) assessed executive functions. The TOL is a classic neuropsychological test for the assessment of executive functions that include planning and problem-solving skills, which has been widely used in diverse cultures [41, 60, 61].

The validation study revealed sound psychometric properties [61]. The TOL consisted of three wooden pegs, which were fixed on a block of wood and three wooden balls of different colors (black, grey and white) that were placed on the pegs and moved from one peg to another. The participants were shown 12 pictures which depicted the TOL with the balls being placed in different positions on the pegs and were asked to arrange the balls to match the positions on the picture. Each trial started from the same starting positions and varied in difficulty due to the number of moves that were allowed to arrange the balls to match the picture (from two to five). Three attempts were granted for each problem. For each problem up to three points could be earned (if successful in the first attempt). The total sum scores ranged from 0 to 36. Higher grades would mean better performance.

### Data analysis

Data were analyzed using SPSS version 23 for Mac. Descriptive statistics were used to compute demographic variables for participants. We z-standardized the two sum scores of PTSD and depressive symptoms severity to compute a mental health problem composite score. Linear regression models were used to test for the association between maltreatment (predictor variable) and the outcome variables of working memory and executive functions. Furthermore, we conducted a simple mediation analysis within the set mediation assumptions that (a) the independent variable would be significantly associated with the dependent variable, (b) the independent variable would be significantly associated with the mediator, and (c) the mediator would be significantly associated with the dependent variable while controlling for the independent variable [62]. These procedures were followed through the analysis process to estimate the mediating role of mental health problems (mediator variable) on the relationship between child maltreatment and the cognitive domains of working memory and executive functions while controlling for age, years of education and trauma load. As test statistic for the mediating role, we used the Sobel test and non-parametric approach of 5000 bootstraps [63]. All models fulfilled the necessary quality criteria for linear regression analysis. The residuals did not deviate significantly from normality, linearity or homoscedasticity and no univariate outliers could be identified. The maximum variance inflation factor did not exceed 1.52. Hence, we did not need to take multicollinearity into consideration.

## Results

Table 1 displays the descriptive statistics for the main study variables. Overall, 232 (100%) of the participant reported to have experienced at least one type of maltreatment in their lifetime. Female participants experienced more maltreatment types than their male counterparts (see Table 1). For example, the majority of participants endorsed having been intentionally pushed by an authority figure (89.7%, n = 208), 89.2% (n = 207) reported having felt that their feelings were not understood by family members. In total, 37.5% of the girls (n = 45,) and 5.4% of the boys (n = 6) indicated that they had been touched in a sexual way (see Additional file 1: Table S1 for more details). The prevalence of PTSD and depression within our sample was 60% (n = 140) and 39% (n = 91), respectively.

### Associations between maltreatment, mental health problems, and executive functions

**Fig. 1:**
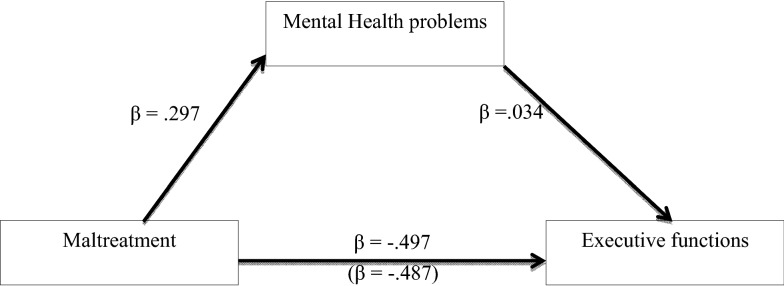
Mediated regression model (*N* = 232) exploring the mediating influence of mental health problems on the relation between child maltreatment and executive functions. This model indicates that the mental health problems did not mediate the association between child maltreatment and executive functions

In a regression model with maltreatment as an explanatory variable and executive functions as the outcome variable while controlling for participant’s age, education in years and trauma load, we found a significant association between maltreatment and executive functions (see Table 2 and Fig. 1). The regression model explained 20% of the variability in executive functions. In addition, trauma load and maltreatment significantly correlated with mental health problems (see Table 2 and Fig. 1). This model explained 26% of the variance of mental health problems. To investigate whether the association between maltreatment and executive functions was mediated by mental health problems, we conducted a simple mediation analysis with maltreatment as an independent variable and executive functions as dependent variable (Fig. 1). When the composite score of mental health problems was added as a mediator variable, the indirect effect of child maltreatment via mental health problems was not significant (see Table 2, Bootstrap results: B = 0.011, SE = 0.023, 95% CI − 0.036, 0.059).

**Table 2:** Association between child maltreatment, mental health and executive functions

	B	SE of B	β	t	95% CI	
					LCI	UCI
Mental health problems^a^						
Age	− 0.017	0.061	− 0.032	− 0.285	− 0.138	0.103
Education in years	0.112	0.080	0.155	1.392	− 0.047	0.270
Trauma	0.276	0.059	0.292	4.715***	0.161	0.392
Maltreatment	0.074	0.015	0.297	4.828***	0.044	0.104
Executive functions^b^						
Age	0.436	0.282	0.179	1.550	− 0.119	0.991
Education in years	− 0.366	0.371	− 0.114	− 0.987	− 1.097	0.365
Trauma	0.427	0.271	0.101	1.577	− 0.106	0.960
Maltreatment	− 0.541	0.071	− 0.487	− 7.657***	− 0.680	− 0.402
Mediation model						
Executive functions^c^						
Age	0.439	0.282	0.180	1.556	− 0.117	0.995
Education in years	− 0.383	0.373	− 0.119	− 1.027	− 1.119	0.352
Trauma	0.385	0.284	0.091	1.354	− 0.175	0.944
Maltreatment^d^	− 0.552	0.074	− 0.497	− 7.428***	− 0.698	− 0.405
Mental health problems	0.152	0.308	0.034	0.494	− 0.455	0.759

	B	SE of B	β	Z	LCI	UCI
Indirect effect^e^						
Maltreatment → mental health problems	0.011	0.023	0.001	0.481	− 0.036	0.059

*B* unstandardized regression weight, *SE* standard error, *β* standardized regression weight, *t* t-test statistic, *CI* confidence interval at 0.05 level of significance ***p  <  0.001

^a^Adj. R^2^ = 0.257; F (4225) = 20.759, *p* < 0.001

^b^Adj. R^2^ = 0.205; F (4225) = 15.799, *p* < 0.001

^c^Adj. R^2^ = 0.203; F (5224) = 12.646, *p* < 0.001

^d^Total effect of maltreatment on executive functions: B = − 0.541, SE of B:0.071, β = − 0.498, t = − 7.657***, LCI: − 0.680, UCI: − 0.402

^e^Sobel test result for indirect effect of maltreatment on executive functions via the mediator mental health problems

### Associations between maltreatment, mental health, and working memory

**Fig. 2:**
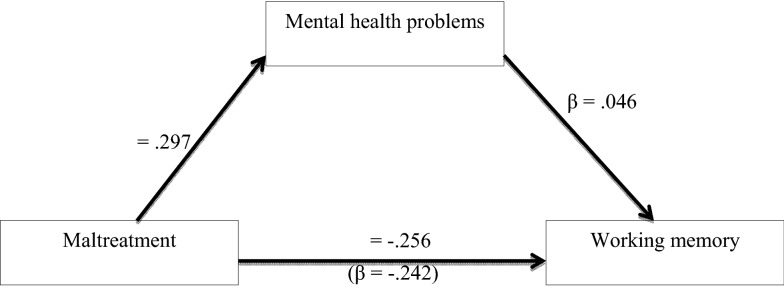
Mediated regression model (*N* = 232) exploring the mediating influence of mental health problems on the relation between child maltreatment and working memory. This model indicates that mental health problems did not mediate the association between child maltreatment and working memory

After controlling for age, years of education, and trauma load, maltreatment was significantly associated with working memory (Table 3 and Fig. 2). The regression model explained 4% of the variability in working memory. Furthermore, maltreatment and trauma load were significantly associated with mental health problems (Table 3 and Fig. 2). The regression model explained 26% of the variations of mental health problems. To investigate whether the association between maltreatment and working memory is mediated by mental health problems, we conducted a simple mediation analysis with maltreatment as independent variable, working memory as dependent variable (Fig. 2). When the mental health composite score was added as a mediator variable, the indirect effect of child maltreatment via mental health problems was not significant (see Table 3, Bootstrap results: B = 0.005, SE = 0.008, 95% CI − 0.010, 0.021).

**Table 3:** Association between child maltreatment, working memory and mental health problems

	B	SE of B	β	t	95% CI	
					LCI	UCI
Mental health problems^a^						
Age	− 0.017	0.061	− 0.032	− 0.285	− 0.138	0.103
Education in years	0.112	0.080	0.155	1.392	− 0.047	0.270
Trauma	0.276	0.059	0.292	4.715***	0.161	0.392
Maltreatment	0.074	0.015	0.297	4.828***	0.044	0.104
Working memory^b^						
Age	− 0.003	0.095	− 0.004	− 0.031	− 0.190	0.184
Education in years	0.111	0.125	0.111	0.884	− 0.136	0.358
Trauma	0.019	0.091	0.014	0.206	− 0.0161	0.199
Maltreatment	− 0.083	0.024	− 0.242	− 3.482***	− 0.130	− 0.036
Mediation model						
Working memory^c^						
Age	− 0.002	0.095	− 0.002	− 0.020	− 0.189	0.186
Education in years	0.104	0.126	0.104	0.822	− 0.145	0.352
Trauma	0.001	0.096	0.001	0.012	− 0.188	0.190
Maltreatment^d^	− 0.088	0.025	− 0.256	− 3.498***	− 0.137	− 0.038
Mental health problems	0.064	0.104	0.046	0.614	− 0.141	0.269

	B	SE of B	β	*Z*	LCI	UCI
Indirect effect^e^						
Maltreatment → mental health problems	0.005	0.008	0.012	0.597	− 0.010	0.021

*B* unstandardized regression weight, *SE* standard error, *β* standardized regression weight, *t* t statistic, *CI* confidence interval at 0.05 level of significance, ***p <  0.001

^a^Adj. R^2^ = 0.257; F (4225) = 20.759, *p* < 0.001

^b^Adj. R^2^ = 0.047; F (4225) = 3.833, *p* = 0.005

^c^Adj. R^2^ = 0.044; F (5224) = 3.124, *p* = 0.01

^d^Total effect of maltreatment on working memory: B = − 0.830, SE of B:0.024, β = − 0.268, t = − 3.482***, LCI: − 0.130, UCI: − 0.036

^e^Sobel test result for indirect effect of maltreatment on working memory via the mediator mental health problems

## Discussion

In this study, we aimed at examining the association between child maltreatment and the cognitive domains of executive functions and working memory as well as the mediating role of mental health problems in a sample of maltreated children and adolescents in Southwestern Uganda. In line with our hypothesis (a) and (b), we found a negative relationship between child maltreatment and both domains of cognitive functioning after controlling for potential influences, such as age, education, and trauma load. However, in contrast to our hypothesis (c), we did not find an indirect effect via mental health problems.

The finding that child maltreatment was negatively associated with executive functions is in line with previous studies suggesting impairments in executive functions among individuals exposed to child maltreatment [29, 64–66]. Furthermore, we found a negative association between child maltreatment and working memory. This observation is also in line with previous findings that found a negative association between child maltreatment and executive functions [13, 67–69]. A possible explanation for these negative associations could be the toxic stress theory that implicates exposure to early childhood adversity in altering the neuro-endocrinal immune system, which renders individuals vulnerable to all forms of functional impairments and disease [15, 18]. For example, children who have experienced harsh punishments and other forms of maltreatment performed poorly on indicators of self-regulation and other cognitive domains [7, 70, 71]. Differences between maltreated children and non-maltreated children can also be seen in children’s physiology, stress response pathways and cortisol levels [72, 73]. With exposure to high levels of maltreatment in our sample, high levels of stress may have negatively impacted hippocampus and the prefrontal cortex functioning. Dense concentration of cortisol receptors in specific brain areas are a possible explanation of cognitive dysfunctioining e.g., in the domains of working memory and executive functions [18, 19, 74]. Based on our own findings and the existing evidence base, including a systematic review [29], we may conclude that children and adolescents exposed to child maltreatment have a higher likelihood of performing poorly on tasks assessing working memory and executive functions. Our findings therefore lend further support to previous research that children exposed to maltreatment risk impairment in cognitive functions [14, 29]. However, it is important to note that most studies on cognitive impairment in individuals exposed to maltreatment are based on cross-sectional data [12, 69, 75, 76]. Therefore, it remains unclear in literature whether maltreatment leads to poor cognitive functions, whether it co-occurs or whether poor functions increase the risk for maltreatment. Future research should focus on longitudinal, prospective, and experimental studies. For example, randomized controlled studies that implement preventative intervention approaches that reduce maltreatment would offer a unique opportunity to test for causal relations [6].

Contrary to our hypothesis, we did not find an indirect effect of child maltreatment via mental health problems on cognitive functions. Although our results are partially in line with one study that also did not find an indirect effect of child maltreatment via PTSD on cognitive functions [12], most studies in high income countries compared individuals with and without mental health problems after trauma exposure [77, 78], while others especially in Africa examined the direct association between mental health problems and cognitive functions [7, 41]. Overall, the current evidence suggests that mental health problems seem to be linked with poor performance in cognitive functioning [79]. In our sample, we could not replicate this finding. Our findings, on the other hand, suggest that it is not the mental health problems but the exposure to maltreatment that is linked to poor performance. As most of the above-mentioned studies did not include maltreatment in their analysis, the question remains whether the found associations would hold when including maltreatment in their analysis. However, it is important to consider that our sample was composed of children and adolescents exposed to severe child maltreatment and the found associations may be specific for severely maltreated children. For example, potential effects on the stress response axis may have played a more prominent role [31, 74]. Due to the limitation of our cross sectional design further research in Africa and Uganda examining the interplay between child maltreatment, mental health problems, and cognitive functions is needed to fully understand the causal mechanisms that lead to cognitive impairments.

### Strength and limitations

Our study examined the relationship between child maltreatment and cognitive functioning and the mediating role of mental health problems among maltreated children and adolescents in Southwestern Uganda. To our knowledge, this is one of the few studies in Africa and other LAMICs that has assessed the mediating role of mental health on child maltreatment and cognitive functions among a sample of maltreated children. Despite our study’s strength, some limitations should be noted: first, the convenience sample does not allow generalizing our findings beyond our specific sample. Secondly, the cross-sectional nature of our study design does not allow us to draw any conclusions about the directionality of our findings. We recommend prospective and experimental studies to shed light on the causal relations. Thirdly, our sample consists of at-risk children. This also limits the possibility to generalize our findings to the general population of children and adolescents. Lastly, it is important to note that biases such as social desirability can never be completely ruled out for subjective reports.

### Conclusion

Our findings therefore provide further support to earlier findings, especially from high income countries, that child maltreatment is associated with poor cognitive functioning. Similarly, based on our findings, we advocate for preventive measures to protect children in Uganda and Africa from violence and maltreatment. Programs aimed at equipping parents, caregivers, and teachers with none violent competencies while interacting with children are highly needed in societies where violence against children is still socially accepted. Based on our findings, we advocate for preventing child maltreatment within schools and families to enable more children in Uganda to grow up in a secure environment and to ensure their healthy development. Future studies in Africa should investigate differential associations and consequences of different types of maltreatment on mental health and cognitive functions.

## Supplementary Information

**Additional file 1: Table S1.** Endorsement of individual items on pediMACE stratified by gender.

## Data Availability

The data sets used and analyzed during the current study are available from the corresponding author on request.

## Abbreviations

PTSD: Post traumatic stress disorder
LMICs: Low- and middle-income countries
CM: Child maltreatment
