# Supplementary material for: Child maltreatment, cognitive functions and the mediating role of mental health problems among maltreated children and adolescents in Uganda
Source: Child Adolesc Psychiatry Ment Health. 2021 Apr 30;15:22. doi: 10.1186/s13034-021-00373-7 (PMC8091686; doi:10.1186/s13034-021-00373-7)
Supplement: Supplementary file 1 — Additional file 1: Table S1. Endorsement of individual items on pediMACE stratified by gender. [file 13034_2021_373_MOESM1_ESM.doc]

| Table S1. Endorsement of individual items on pediMACE stratified by gender | | | | | | | | | |
| --- | --- | --- | --- | --- | --- | --- | --- | --- | --- |
|  | Total (232) | | Male (112) | | Female(120) | | |  |  |
| Items | n | % | n | % | n | % | X^2^ | | p |
| Having been intentionally pushed you | 208 | 89.7 | 105 | 93.8 | 103 | 85.8 | 3.915 | | 0.048 |
| Parental figure not understanding feelings | 207 | 89.2 | 96 | 85.7 | 111 | 92.5 | 2.774 | | 0.096 |
| Having been called names or hurting full things (family member) | 206 | 88.8 | 97 | 86.6 | 109 | 90.8 | 1.04 | | 0.308 |
| Having been spanked with a palm | 203 | 87.5 | 101 | 90.2 | 102 | 85 | 1.420 | | 0.233 |
| Having been spanked with an object | 200 | 86.2 | 99 | 88.4 | 101 | 84.2 | 0.870 | | 0.351 |
| Having been yelled or screamed at | 199 | 85.8 | 96 | 85.7 | 103 | 85.8 | 0.001 | | 0.979 |
| Witnessing adults at home arguing | 193 | 83.2 | 92 | 82.1 | 101 | 84.2 | 0.170 | | 0.680 |
| Witnessing sibling being pushing | 193 | 83.5 | 90 | 80.4 | 103 | 86.6 | 1.612 | | 0.204 |
| Witnessing sibling being hit with a palm | 186 | 80.2 | 86 | 76.8 | 100 | 83.3 | 1.562 | | 0.211 |
| Being taken care of by a family member | 182 | 78.4 | 86 | 76.8 | 96 | 80 | 0.354 | | 0.552 |
| Having won dirty clothes | 180 | 77.6 | 92 | 82.1 | 88 | 73.3 | 2.585 | | 0.108 |
| Having had not enough food to eat | 179 | 77.2 | 88 | 78.6 | 91 | 75.8 | 0.246 | | 0.620 |
| A family member brought you to the doctor | 173 | 74.6 | 75 | 67 | 98 | 81.7 | 6.603 | | 0.010 |
| Having been made feel loved | 173 | 74.6 | 90 | 80.4 | 83 | 69.2 | 3.825 | | 0.050 |
| Anybody said hurtful things to me | 172 | 74.1 | 88 | 78.6 | 84 | 70 | 2.220 | | 0.136 |
| Anybody called you or said hurtful things | 152 | 65.5 | 64 | 57.1 | 88 | 73.3 | 6.722 | | 0.010 |
| Either mother or father passed away | 151 | 65.1 | 75 | 67 | 76 | 63.3 | 0.336 | | 0.562 |
| Witnessed adults other pushing you mother | 150 | 64.7 | 70 | 62.5 | 80 | 66.7 | 0.440 | | 0.507 |
| Anybody said things behind my back | 150 | 60.7 | 68 | 60.7 | 82 | 68.9 | 1.701 | | 0.192 |
| Anybody yelled screamed at you | 142 | 61.2 | 63 | 56.2 | 79 | 65.8 | 2.241 | | 0.134 |
| Family member helped with home work | 136 | 58.6 | 66 | 58.9 | 70 | 58.3 | 0.008 | | 0.927 |
| Anybody intentionally pushed or kicked you | 131 | 56.5 | 57 | 50.9 | 74 | 61.7 | 2.735 | | 0.098 |
| Spanked with the palm | 129 | 55.6 | 56 | 50 | 73 | 60.8 | 2.754 | | 0.097 |
| Having been spanked with an object | 117 | 50.4 | 55 | 49.1 | 62 | 51.7 | 0.152 | | 0.697 |
| Anybody excluded me from activities | 116 | 50 | 51 | 45.5 | 65 | 54.2 | 1.726 | | 0.189 |
| Witnessed adults pushing my father | 101 | 43.7 | 47 | 42.0 | 54 | 45.4 | 0.273 | | 0.601 |
| Witnessed adults hitting mother | 93 | 40.6 | 41 | 36.9 | 52 | 44.1 | 1.206 | | 0.272 |
| Being locked in closet narrow and dark place | 79 | 34.1 | 49 | 43.8 | 30 | 25 | 9.069 | | 0.003 |
| Witnessed anybody hitting your sibling to injury | 78 | 33.6 | 32 | 28.6 | 46 | 38.3 | 2.474 | | 0.116 |
| Parents separated or divorced | 75 | 32.3 | 32 | 28.6 | 43 | 35.8 | 1.396 | | 0.237 |
| Anybody touched my body in an appropriate way | 57 | 24.6 | 27 | 24.1 | 30 | 25.0 | 0.025 | | 0.875 |
| Anybody touched my body in an in appropriate way | 51 | 22 | 6 | 5.4 | 45 | 37.5 | 34.899 | | <0.001 |
| Anybody made me touch her body in appropriately | 47 | 20.3 | 6 | 5.4 | 41 | 34.5 | 30.140 | | <0.001 |
| Anybody hit me so hard to injury | 46 | 19.9 | 30 | 26.8 | 16 | 13.4 | 6.438 | | 0.011 |
| Anybody entered anything into my body | 41 | 17.7 | 2 | 1.8 | 39 | 32.5 | 37.562 | | <0.001 |
| Witnessed anybody touching your siblings’ body | 33 | 14.2 | 12 | 10.7 | 21 | 17.5 | 2.186 | | 0.139 |
| Witnessed adults at home touching mothers’ body | 29 | 12.5 | 16 | 14.3 | 13 | 10.8 | 0.631 | | 0.427 |
| Witnessed adults at home hitting you father | 28 | 12.1 | 12 | 10.7 | 16 | 13.3 | 0.374 | | 0.541 |
| Anybody locked you in a narrow and dark place | 26 | 11.2 | 14 | 12.5 | 12 | 10 | 0.364 | | 0.546 |
| Witnessed your sibling being made to touch some body | 18 | 7.8 | 7 | 6.4 | 11 | 9.2 | 0.625 | | 0.429 |
| Witnessed anybody entering any part of your siblings’ body | 11 | 4.8 | 4 | 3.6 | 7 | 5.8 | 0.632 | | 0.427 |
| Witnessed adults living at home touching fathers’ body | 8 | 3.4 | 4 | 3.6 | 4 | 3.3 | 0.010 | | 0.921 |
| Anybody entered an object in my body | 6 | 2.6 | 1 | 0.9 | 5 | 4.2 | 2.461 | | 0.117 |
